# Isolation and characterization of probiotics from dairies

**Published:** 2017-08

**Authors:** Babak Haghshenas, Yousef Nami, Ali Almasi, Norhafizah Abdullah, Dayang Radiah, Rozita Rosli, Abolfazl Barzegari, Ahmad Yari Khosroushahi

**Affiliations:** 1 Departmen of Food Biotechnology, Branch for Northwest & West Region, Agricultural Biotechnology Research Institute of Iran, Agricultural Research, Education and Extension Organization (AREEO), Tabriz, Iran; 2 University of Medical Sciences, Department of Health and Social Medicine, Kermanshah, Iran; 3 Chemical and Environmental Engineering Department, Faculty of Engineering, University Putra Malaysia, 43400 UPM Serdang, Selangor, Malaysia; 4 Institute of Biosciences, University Putra Malaysia, 43400 UPM Serdang, Selangor, Malaysia; 5 Biotechnology Research Center, Department of Pharmacognosy, Faculty of Pharmacy, Tabriz University of Medical Sciences, Tabriz, Iran

**Keywords:** Fingerprinting, Probiotic, Dairy products, Cheese, Shiraz

## Abstract

**Background and Objectives:**

Probiotics are live microorganisms, which show beneficial health effects on hosts once consumed in sufficient amounts. LAB group can be isolated and characterized from traditional dairy sources. This study aimed at isolating, identifying, and *in vitro* characterizing (low pH/high bile salt tolerance, antibacterial activity, and antibiotic susceptibility) LAB strains from traditional Iranian dairy products.

**Materials and Methods:**

Isolated strains were identified by Gram staining, catalase assay, and 3 molecular identification methods; namely, (GTG) 5-PCR fingerprinting, ARDRA, and 16S rDNA gene sequencing.

**Results:**

A total of 19 LAB strains belonging to 4 genera (*Lactococcus, Leuconostoc, Lactobacillus* and *Enterococcus*) were identified.

**Conclusion:**

The experiments revealed that *L. plantarum* 15HN, *L. lactis* subsp. *cremoris* 44L and *E. mundtii* 50H strains, which were isolated from shiraz, cheese and shiraz, respectively, displayed a desirable tolerance to low pH and high bile salts, favorable anti-pathogen activity, and acceptable antibiotic susceptibility; hence, they could be considered as novel probiotic candidates and applied in the food industry.

## INTRODUCTION

Diverse traditional dairy products with health-enhancing benefits, such as improvement of nutrient absorption, inactivation of toxins and anti-pathogenic activities, are used worldwide (1). Traditional dairy products, such as curd, tarkhineh, shiraz, yogurt, and cheese, are produced in different countries, particularly in Iran. Shiraz is a tasty and authentic traditional dairy product in this region. It is prepared out of buttermilk or yogurt with some herbal plants, such as nigella and fennel. Meanwhile, tarkhineh is a traditional cereal product made of a mixture of fermented milk, yogurt, cooked vegetables (turnip) with some grains such as cracked wheat. These dairy products are consumed in this region and exported to other regions, including Europe, as a result of increasing demands (2).

Probiotics are microorganism’s active factors, which show the beneficial impacts on the host health. Probiotics significantly affect the bioavailability of nutrients in the human body by facilitating the absorption of magnesium and calcium from milk proteins, digesting lactose and producing folate and B vitamins. *Lactobacillus* and *Enterococcus* species are common lactic acid bacteria (Gram-positive and non-toxic bacteria), which are usually consumed as probiotics (3). Lactic acid bacteria (LAB) are generally recognized as safe microorganisms. Most of probiotics include LAB group, which is isolated from safe sources, such as fermented dairy products and can be introduced as probiotics.

LAB grows under similar conditions. Thus, these bacteria could not be identified and differentiated by traditional phenotyping and biochemical methods, such as sugar fermentation at a genus level, as these techniques do not provide clear classification results. Rapid, accurate, and practical molecular identification techniques, such as 16S rDNA sequencing and analyzing (4), specific gene sequencing, ribotyping with specific probes, Repetitive Bacterial DNA Elements PCR (REP-PCR), Pulsed-Field Gel Electrophoresis (PFGE), Denaturing Gradient Gel Electrophoresis (DGGE), Randomly Amplified Polymorphic DNA (RAPD), and Amplified Ribosomal DNA Restriction Analysis (ARDRA), have been designed to identify isolated bacteria at genus, species, or subspecies levels (5). However, when these methods are used individually, they fail to produce strong clustering results at strain levels, thus, an effective combination of these techniques should be considered. Therefore, this study aimed at screening traditional dairy products of the west of Iran microbiota to determine new strains with high probiotic capability by employing important morphological and biochemical assays with 3 molecular methods; namely, 16S rDNA sequencing, REP-PCR (GTG-PCR) and ARDRA.

## MATERIALS AND METHODS

### Sampling and isolation.

A total of 200 samples of traditional dairy products including cheese, yogurt, curd, shiraz and tarkhineh from west of Iran were prepared. Five grams of each dairy sample was suspended in 2% w/v sodium citrate solution and homogenized using Stomacher 400 Circulator (Seward Laboratory Systems Inc, USA) for 2 minutes. One mL of the samples was added to 24 mL of MRS broth (Merck, Germany) in anaerobic conditions containing 5% CO_2_. These diluted solutions (0.02 mL) were spread on MRS agar plates (Merck, Germany) and incubated for 48 hours. The single colonies on the growth agar plate were selected and transferred to 15 mL of broth culture medium for 24 hours at 37°C. The isolates were stored in 30% (w/v) glycerol and 10% (w/v) skim milk at −70°C for further assessments (6).

### 16S rDNA fragment amplification and sequencing.

In this study, 16S rDNA amplification and sequencing was performed based on methodology described previously by Haghshenas et al. 2014 (7).

### ARDRA analysis.

The, 16S rDNA amplified products were digested using *Pst* I restriction enzyme (Fermentas, St. Leon-Rot, Germany) for 2 hours at 37°C. The digestion reaction contained 5 units of *Pst* I enzyme, 2 μL of 10× buffer and 5 μL of PCR product. The digested products were electrophoresed on 1.5% agarose gel at voltage of 70 V for 1 hour and visualized by utilizing SYBR Green dye (DNA safe stain, Tehran, Iran).

### (GTG)_5_-PCR fingerprinting and data analysis.

In this study, (GTG)_5_-PCR amplification was performed according to method described in previous study (8).

### Low pH and high bile salt tolerance assessment.

The isolated strains were screened to select the cells resistant to low pH condition according to Nami et al. (9).

Resistant bacteria were determined based on high bile concentration according to the method of Haghshenas et al. 2016 (10).

### Antimicrobial Activity.

The modified well diffusion method was used to determine the antibacterial activities of isolated bacteria (7). Antibacterial activity assessments were conducted against clinically important human pathogens (Table 1). Based on the diameter of the inhibition zone, anti-pathogen activities were divided into strong (diameter ≥ 20 mm), moderate (20 mm ≤ diameter ≥ 10 mm) and weak (diameter ≤ 10 mm) (11).

**Table 1.** Sequencing results of 19 representative isolated LAB and their dairy origin and survival rates of isolated LAB after 3 h incubation at pH 2.5 and 0.3% bile salts

**Isolates**	**Sequencing Results**	**Actual Sequencing Homology (%)**	**Dairy Origen**	**SR (%) in pH 2.5**	**SR (%) in high bile salt**
10H2	*Leuconostoc mesenteroides* subsp. *cremoris*	99.4	yogurt	43	65
39C	*Enterococcus durans*	100	yogurt	82	96
46Lac	*Lactobacillus paracasei* subsp. *paracasei*	100	yogurt	76	92
11H	*Lactococcus lactis* subsp. *cremoris*	100	yogurt	44	65
41Lac	*Leuconostoc mesenteroides* subsp. *mesenteroides*	99.6	curd	84	90
13C	*Enterococcus faecalis*	99.3	curd	73	98
13H2	*Lactococcus lactis* subsp. *lactis*	100	curd	53	71
13H	*Lactococcus lactis* subsp. *lactis*	100	curd	66	82
18H	*Leuconostoc mesenteroides* subsp. *mesenteroides*	99.8	tarkhineh	53	74
35C	*Leuconostoc mesenteroides* subsp. *mesenteroides*	99.2	tarkhineh	47	70
44Lac	*Lactococcus lactis* subsp. *lactis*	99.5	cheese	85	94
44L	*Lactococcus lactis* subsp. *cremoris*	100	cheese	81	95
2H2	*Leuconostoc mesenteroides* subsp. *mesenteroides*	99.7	cheese	61	80
19H2	*Leuconostoc mesenteroides* subsp. *cremoris*	100	cheese	55	77
5H	*Leuconostoc lactis*	100	cheese	49	66
15H	*Leuconostoc lactis*	99.3	shiraz	64	82
50H	*Enterococcus mundtii*	100	shiraz	78	98
50H2	*Enterococcus mundtii*	99.8	shiraz	51	70
15HN	*Lactobacillus plantarum*	99.0	shiraz	71	88

SR: Survival Rate

### Antibiotic susceptibility.

Disc diffusion method was used to determine the antibiotic susceptibility of each isolated strain. After spreading each strain in anaerobic condition for 24 hours on Mueller-Hinton agar plates, antibiotic disks (Padtan Teb Co., Tehran, Iran) were placed on the plates using sterilized forceps. These plates were incubated for 24 hours at 37°C. Next, the clear zones around each disc were measured.

## RESULTS

### Morphological and biochemical assays.

A total of 92 hemispherical white or achromatic colonies were grown on related culture media. Each colony was separately propagated for further assessments. Among these Gram-positive and catalase-negative strains, 25 strains were isolated from cheese, 22 from curd, 17 from yogurt, 16 from shiraz, and 12 from tarkhineh.

### Identification by 16S rDNA sequencing.

The PCR-amplified 1500 bp fragments of 16S rDNA gene of the isolates were sequenced. Sequences were blasted with the deposited sequences in GenBank. Isolates with 99% to 100% homology were identified by considering the threshold values of taxonomical studies (97%) (12). These 19 strains belonged to 4 genera (*Lactococcus, Leuconostoc, Lactobacillus,* and *Enterococcus*) (Table 1). The maximum and minimum LAB populations across the LAB isolates from 5 traditional dairy products (yogurt, cheese, curd, shiraz, and tarkhineh) belonged to *Leuconostoc* (42%) and *Lactobacillus* (11%).

### Clustering by ARDRA.

**Fig. 1.**
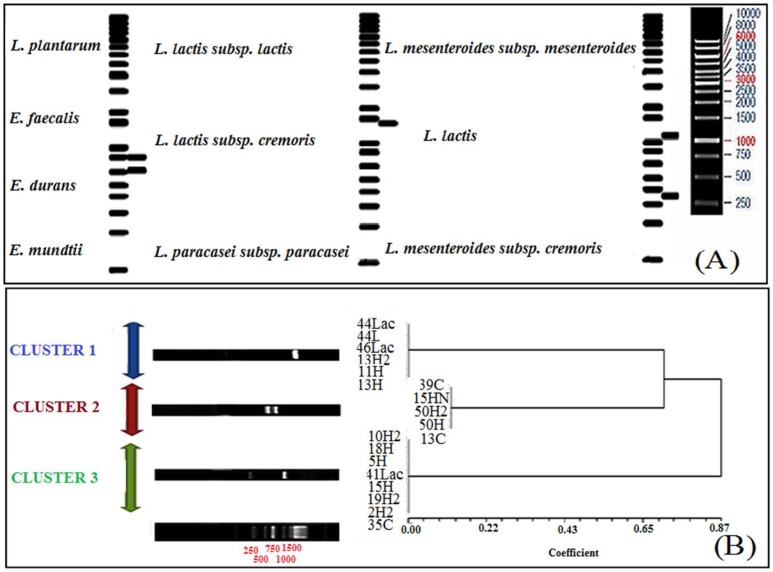
The ARDERA analysis results. (A) the virtually cleaving pattern of standard bacteria species in GenBank as a reference species by using the *Pst* I enzyme and (B) three distinct groups obtained by ARDRA clustering with higher homology to *Lactobacillus, Leuconostoc, Lactococcus* and *Enterococcus* strains

The amplified 16S rDNA from 19 isolated strains were restricted by *Pst* I restriction enzyme. Three different restricted patterns were observed after performing on 1.5% agarose gel. In the first pattern (Fig. 1, cluster I), *Pst* I enzyme failed to restrict the amplified products and only 1 fragment with approximately 1500 bp was observed in the gel; 2 fragments with the sizes of 650 and 850 bp were seen in the second pattern (Fig. 1. cluster II); and 2 fragments with the sizes of 350 and 1150 bp were found in the third pattern (Fig. 1. cluster III). The results revealed 1 restriction site; 3 distinct clusters were obtained by analyzing these polymorphic patterns with NTSYS-PC (Fig. 1 (B)).

Then, 6 isolated strains were grouped into Cluster 1, 5 isolates into Cluster 2 and 8 isolates into Cluster 3. To predict and identify the members of each cluster, the 16S rDNA sequences of each candidate strain in GenBank were selected according to the sequencing patterns virtually restricted using *Pst* I restriction enzyme in ApE A plasmid editor software. Fig. 1 panel (A) demonstrates virtually restricted patterns, which can be divided into 3 clusters. The first cluster includes *Lactococcus* and some *Lactobacillus* species, such as *L. paracasei*. The second cluster comprises the *Enterococcus* genera and some *Lactobacillus* species, such as *L. plantarum* and the third cluster covers *Leuconostoc* species. Discrimination at the genus level was performed by comparing the experimental and virtually restricted patterns.

### Clustering by (GTG)_5_-PCR.

**Fig. 2.**
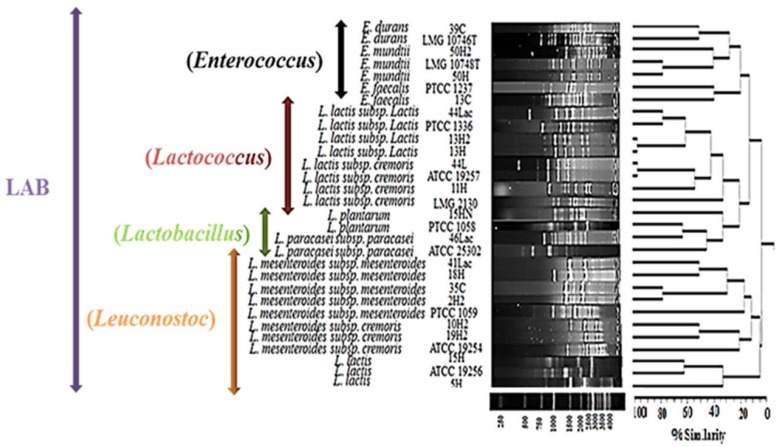
Dendrogram generated after cluster analysis of the digitized (GTG)_5_-PCR fingerprints of a total of 11 reference strains and 19 LAB isolates. The dendrogram was constructed using the un-weighted pair-group method using arithmetic averages with correlation levels expressed as percentage values of the Pearson correlation coefficient. ATCC: American Type Culture Collection, Manassas, VA, USA; LMG: Laboratorium voor Microbiologie, Universiteit Gent, Gent, Belgium; PTCC: Persian Type Culture Collection, Iranian Research Organization for Science and Technology (IROST), Tehran, Iran. T: type strain.

The GTG-primer yielded the lowest number of bands from 4 to a maximum of 16 visualized PCR products with an average of 8. Polymorphic band patterns, ranging from 300 bp to 4000 bp, were assessed using NTSYS-PC. The isolated strains were reanalyzed to verify the reproducibility of the (GTG)_5_-PCR fingerprinting. All strains provided the same band patterns without qualitative differences as a result of missing bands. However, differences in the band intensity of several fingerprints were observed. Meanwhile, the validation of clustering results was confirmed by comparing with the 16S rDNA sequencing results and ARDRA experimental/virtual band patterns (Fig. 2).

According to (GTG)_5_-PCR fingerprinting results, all isolates were grouped in 4 separated clusters (genus level) according to their respective taxonomic designations. The first cluster was identified as *Enterococcus* genus with 7 strains; the second cluster was identified as *Lactococcus* genus with 8 strains; *Lactobacillus* genus with 4 strains was classified as the third cluster; and the last cluster was identified as *Leuconostoc* genus with 11 strains. Moreover, they were clearly grouped into well-separated clusters representing single species. Four strains were separated into a cluster representing *L. mesenteroides* subsp. *mesenteroides*; 3 strains clustered with *L. lactis* subsp. *lactis*; 2 strains matched *E. mundtii, L. lactis* subsp. *cremoris, L. mesenteroides* subsp. *cremoris* and *L. lactis*; and 1 strain showed fingerprints similar to *E. durans, E. faecalis, L. plantarum* and *L. paracasei* subsp. paracasei. In short, discrimination at the species level was performed by comparing the (GTG)_5_-PCR band patterns of isolates and reference strains (Fig. 2).

### Low pH and high bile salt tolerance assessment.

The survival rates of 19 isolated LAB strains after 3 hours of incubation at pH 2.5 are presented in Table 1. Based on the results, all 19 selected strains retained their viability even after 3 hours of exposure to pH 2.5. Notably, a broad variation in survival was observed at this condition. The moderate survival rates, ranging from 71% to 76%, were observed in *Lactobacillus* strains, whereas the survival rates of *Lactococcus* and *Leuconostoc* strains ranged from 43% to 85%. Moreover, *Enterococcus* strains showed high tolerance, ranging from 51% to 82%, to acidic conditions. The strains with the most efficient tolerance to acidic conditions were *L. lactis* subsp. *lactis* 44Lac, *L. mesenteroides* subsp. *mesenteroides* 41Lac, *E. durans* 39C, and *L. lactis* subsp. *cremoris* 44L, with survival rates of 85%, 84%, 82% and 81%, respectively. Meanwhile, 8 out of 19 isolated strains, showed high tolerance to acidic conditions (survival rates > 70%); this tolerance was, however, strain specific. The survival rates of 19 isolated LAB in 0.3% oxgall are displayed in Table 1. All the isolated strains displayed high tolerance to bile salt conditions, ranging from 6% to 25% higher than low pH tolerance. Moreover, tolerance to a high bile salt condition was also strain specific.

The survival rates of *Lactobacillus* strains ranged from 88% to 92%, whereas the survival rates of *Lactococcus* and *Leuconostoc* strains ranged from 65% to 95%. *Enterococcus* strains revealed high resistance to high concentrations of bile salts, ranging from 70% to 98%. Meanwhile, 8 out of 19 isolates revealed high survival rates, with >86%, under high bile conditions. The strains with the highest tolerance to 0.3% oxgall were *E. mundtii* 50H, *E. faecalis* 13C, and *E. Durans* 39C, with the survival rates of 98%, 98%, and 96%, respectively.

Isolates 46Lac, 15HN, 41Lac, 44Lac, 44L, 13C, 50H, and 39C showed high survival rates under low pH (>70%) and high bile conditions (>86%). Consequently, these 8 strains were selected for further probiotic analysis.

### Antimicrobial Activity.

Table 2 shows that 8 isolated strains (*L. paracasei* 46Lac, *L. plantarum* 15HN, *L. mesenteroides* 41Lac, *L. lactis* 44Lac, *L. lactis* 44L, *E. faecalis* 13C, *E. mundtii* 50H and *E. durans* 39C) displayed significant anti-pathogenic activities against indicator microorganisms.

**Table 2.** The inhibitory effect of isolated strains against pathogenic microorganisms

**Pathogens**	Diameter of inhibition zone (mm)							

	**46Lac**	**15HN**	**41Lac**	**44Lac**	**44L**	**13C**	**50H**	**39C**
*P. aeruginosa*	12.3±1.2	11.3±0.3	0.0±0.0	0.0±0.0	14.0±1.0	15.3±0.7	16.7±0.3	15.0±0.6
*C. albicans*	0.0±0.0	10.0±0.0	0.0±0.0	0.0±0.0	10.3±0.3	0.0±0.0	13.0±0.0	0.0±0.0
*S. marcesens*	11.7±0.3	17.3±0.0	0.0±0.0	12.3±1.2	15.7±0.3	13.0±0.6	14.0±1.0	11.0±0.0
*E. faecalis*	0.0±0.0	0.0±0.0	0.0±0.0	0.0±0.0	12.0±0.0	0.0±0.0	12.3±1.2	0.0±0.0
*S. saprophyticus*	0.0±0.0	11.3±0.7	13.3±0.3	0.0±0.0	0.0±0.0	14.0±0.6	0.0±0.0	0.0±0.0
*S. mutans*	12.0±0.0	17.3±0.6	0.0±0.0	0.0±0.0	0.0±0.0	0.0±0.0	0.0±0.0	12.3±1.2
*E. coli (0157)*	14.7±1.2	10.0±0.0	0.0±0.0	12.0±0.6	12.0±1.0	0.0±0.0	13.3±0.3	12.0±0.0
*S. typhimurium*	0.0±0.0	12.3±1.2	0.0±0.0	0.0±0.0	11.3±1.2	0.0±0.0	13.3±0.3	0.0±0.0
*S. aureus*	0.0±0.0	11.7±0.3	0.0±0.0	0.0±0.0	14.7±0.3	0.0±0.0	13.0±0.0	13.7±1.2
*E. coli (026)*	0.0±0.0	12.3±0.7	0.0±0.0	13.7±1.2	13.3±0.6	0.0±0.0	15.7±0.3	0.0±0.0
*B. cereus*	0.0±0.0	10.0±0.0	0.0±0.0	0.0±0.0	0.0±0.0	0.0±0.0	0.0±0.0	0.0±0.0
*L. monocytogenes*	0.0±0.0	13.7±0.9	0.0±0.0	0.0±0.0	15.7±0.7	0.0±0.0	17.0±0.0	0.0±0.0
*K. pneumoniae*	13.3±0.7	12.0±0.6	0.0±0.0	12.7±0.3	12.3±0.7	12.7±1.2	13.3±1.2	13.0±1.0
*S. flexneri*	11.0±0.0	12.0±0.0	0.0±0.0	0.0±0.0	11.0±0.0	11.0±0.0	14.0±0.6	11.3±0.7

Notes: values are mean ± standard error

S (strong *r* ≥20 mm), M (moderate *r*<20 mm and >10 mm), and W (weak ≤10 mm)

46Lac: (*L. paracasei* subsp. *paracasei*); 15HN: (*L. plantarum*); 41Lac: (*L. mesenteroides* subsp. *mesenteroides*); 44Lac: *L. lactis* subsp. *lactis*); 44L (*L. lactis* subsp. *cremoris*); 13C (*E. faecalis*); 50H (*E. mundtii*); 39C (*E. durans*).

*Lactobacillus* species, particularly *L. plantarum* 15HN, showed the most efficient antagonistic activity and inhibited the growth of 13 indicator pathogens among the isolated bacteria. Meanwhile, *L. lactis* 44L and *E. mundtii* 50H exhibited an overall good antagonistic activity and inhibited the growth of indicator pathogens.

### Antibiotic susceptibility.

The antibiotic susceptibility results of 8 isolated LAB against clinically important antibiotics are presented in Table 3. Based on our findings, all 8 isolated bacteria were sensitive or semi-sensitive to tetracycline and clindamycin. *Lactobacillus* and *Enterococcus* strains generally displayed the highest susceptibility to the majority of antibiotics. Moreover, *L. paracasei* subsp. *paracasei* 46Lac and *E. durans* 39C, which were isolated from yogurt, displayed the best results and were sensitive or semi-sensitive to all antibiotics. On the other hand, isolated *E. faecalis* 13C from curd, resistance to 5 antibiotics (erythromycin, ampicillin, vancomycin, chloramphenicol and penicillin), was the most resistant isolate. The maximum resistance to vancomycin was observed among the isolates.

**Table 3.** Antibiotic susceptibility of isolated LAB against the high consumption antibiotics by disc diffusion assay

**Isolated Strains**	Diameter of inhibition zone (mm)								

	C	TE	ER	AM	GE	CC	SLX	P	V
*L. paracasei* subsp. *paracasei* 46Lac	30S	30S	30S	30S	30S	30S	30S	30S	30S
*L. plantarum* 15HN	20S	20S	20S	20S	20S	20S	20S	20S	20S
*L. mesenteroides* subsp. *mesenteroides* 41Lac	23S	23S	23S	23S	23S	23S	23S	23S	23S
*L. lactis* subsp. *lactis* 44Lac	28S	28S	28S	28S	28S	28S	28S	28S	28S
*L. lactis* subsp. *cremoris* 44L	14I	14I	14I	14I	14I	14I	14I	14I	14I
*E. faecalis* 13C	0R	0R	0R	0R	0R	0R	0R	0R	0R
*E. mundtii* 50H	18S	18S	18S	18S	18S	18S	18S	18S	18S
*E. durans* 39C	22S	22S	22S	22S	22S	22S	22S	22S	22S

C: chloramphenicol; TE: tetracycline; ER: erythromycin; AM: Ampicillin; GE: gentamycin; CC: clindamycin; SLX: sulfamethoxazol; P: penicillin; V: vancomycin

Erythromycin results based on R ≤13 mm; I: 13–23 mm; S≥23 mm.

Gentamycin results based on R ≤6 mm; I: 7–9 mm; S≥10 mm.

Vancomycin results based on R ≤12 mm; I: 12–13 mm; S≥13 mm.

I: intermediate (zone diameter, 12.5–17.4mm); R: resistant (zone diameter, ≤12.4mm); S: susceptible (zone diameter, ≥17.5).

## DISCUSSION

Based on FAO/WHO guidelines, analyzing and identifying probiotic microorganisms with 16S rDNA patterns can be considered as an accessible, cost-effective and suitable technique compared with other costly and time-consuming molecular techniques (13). This technique has also been utilized as an effective method to analyze and isolate lactic acid bacteria and acetic acid bacteria in *Lactobacillus*, *Leuconostoc* and *Acetobacter* genera, which were isolated from fermented dairy products (14). However, based on the results and in comparison with the deposited sequences in GeneBank of NCBI site, the homology levels among some strains in LAB group (*L. lactis* subsp. *lactis* 13H2 and *L. lactis* subsp. *lactis* 13H) are suggested to be more than 99% (15). This indicates that the 16S rDNA sequencing technique is not validated or sufficient enough for discrimination at strain levels. For differentiation at strain level, the sequencing results were compared with (GTG)_5_-PCR band patterns and ARDRA results.

On the other hand, ARDRA is an accurate and rapid technique that can be used to differentiate isolates at a genus level. In this method, different restriction enzymes, such as *Bfa* I, *Mse* I, *Fse* I and *Alu* I, were previously used to distinguish between LAB and acetic acid bacteria (16). Contrary to our results, the discrimination power of ARDRA was very low. This technique cannot generate reliable discriminative results, even at the genus level of some closely related genera such as *Lactococcus*.

Meanwhile, (GTG)_5_-PCR fingerprinting method with a high ability power can be used to form clearly distinguishable patterns. (GTG)_5_-PCR fingerprinting is an effective method to analyze lactic acid bacteria/acetic acid bacteria isolated from fermented dairy products (14). This method is also more reproducible than RAPD because higher annealing temperatures and longer primers are used in the former than in the latter. The high discriminative power of (GTG)_5_-PCR fingerprinting on some bacterial species, such as *Salmonella* and *E. coli* strains, has been previously reported in different studies (17). However, it requires a large collection of reference strains, which makes it laborious and costly (14). Due to the low variety of reference strains in LAB group (18), they were selected based on sequencing results. Moreover, in this study, due to the low genetic similarity levels and high diversity (different genera in LAB group), the banding patterns were not solely discriminative. Then, the validation of (GTG)_5_-PCR fingerprinting method was verified by comparing (GTG)_5_-PCR clustering results with ARDRA experimental/virtual band patterns (Fig. 1) and 16S rDNA sequencing results (Table 1). These 19 separated band patterns verified that each isolate belongs to different LAB strains. Meanwhile, results from combined 16S rDNA sequencing with (GTG)_5_-PCR results revealed that all 19 isolates from Iranian dairy products were well-characterized and identified until the strain level by 16S rDNA sequencing method (Fig. 2). However, this result could not be achieved when only 1 technique was used. Our findings confirmed that this combined method can be used as an accessible, low-cost and suitable technique to identify lactic acid bacteria from dairy products until the strain level.

Health-improving effects of probiotics include resistance to gastrointestinal acid and bile, having high anti-microbial activities and susceptibility against antibiotics. Therefore, probiotic characterization must be performed through standard *in vitro* experiments.

Probiotic strains must tolerate inverse conditions (ie, low pH [pH 2.0 to pH 3.0] and high bile salts [0.3% (w/v)]) for a minimum of 90 minutes (19). In this study, 8 out of 19 isolated strains displayed high survival rates under low pH (>71 %) and high bile salt conditions (>88%). However, *Enterococcus* strains showed better low pH tolerance than others. This high tolerance capability is related to the bilayer membrane structure, which enables easy tolerance of inverse conditions (19). These results are in contrast with other studies that found that *Lactobacillus* strains tolerate inverse conditions better than other genera among LAB isolates.

The effects of bile salts on bacterial probiotic cells differed under acidic conditions. The resistance of probiotics to bile salts can be unpredictable and higher than that of the acid tolerance patterns (20). Similar to our findings, the results in other studies have revealed that *Enterococcus* strains are more tolerant to high bile salt conditions than other LAB strains (21).

*Lactobacillus* and *Enterococcus* strains, in comparison with other isolated bacteria, displayed better anti-pathogenic activity. Our findings were supported by evidence showing the high anti-microbial activities of *Lactobacillus* and *Enterococcus* strains on diverse pathogenesis due to secretion of bacteriocins, biosurfactants, H_2_O_2_, and organic acids (22). Moreover, Gram-negative pathogens, such as *S. marcesens* (PTCC 1187) and *K. pneumoniae* (PTCC 1053), were more sensitive than Gram-positive pathogens, such as *B. cereus* subsp. *kenyae* (PTCC 1539) and *E. faecalis* (PTCC 1394) (Tables 2).

The sensitivity of probiotics to conventional antibiotics is a fundamental health-promoting characteristic. The overuse of antibiotics can spread resistance genes across a region and transfer these genes to other microbial communities (23).

All isolates were sensitive or semi-sensitive to tetracycline and clindamycin, hence, these antibiotics can be used in their selective growth media but re-establishment of probiotic balance in the gut tract must be considered after tetracycline and clindamycin treatment. The sensitivity to these antibiotics probability was due to limited usage of antibiotics in the rural area of Iran (Kermanshah province). The high resistance to tetracycline among the probiotic bacteria was reported by other researches. On the other hand, in spite of limited reports on clindamycin resistance genes among probiotics (24), these resistance genes can be easily transferred to the pathogenic strains, such as *S. aureus* (25). The maximum resistance was observed for vancomycin and a similar result was also observed by other researches. Vancomycin is one of the last antibiotics, which is highly effective against clinical infections caused by multidrug-resistant pathogens; thus, resistance against vancomycin is critical among probiotics. However, the specific isolated LAB including strains of *L. paracasei* subsp. *paracasei* 46Lac, *L. lactis* subsp. *lactis* 44Lac, *L. lactis* subsp. *cremoris* 44L, *E. mundtii* 50H and *E. durans* 39C were sensitive to vancomycin.

## CONCLUSION

The combination of (GTG)_5_-PCR fingerprinting method and ARDRA and the 16S rDNA gene sequencing with high discriminative power can be used as an effective, low-cost and rapid alternative to identify and differentiate dairy and non-dairy-associated LAB. Findings indicated that *L. plantarum* 15HN, *L. lactis* subsp. *cremoris* 44L and *E. mundtii* 50H strains, which were isolated from shiraz, cheese and shiraz, respectively, displayed a desirable tolerance to low pH and high bile salts, favorable anti-pathogen activity, and acceptable antibiotic susceptibility; hence, these bacteria may be used as probiotics.

## References

[1] Mathara JM Schillinger U Kutima PM Mbugua SK Guigas C Franz C Holzapfel WH Functional properties of *Lactobacillus plantarum* strains isolated from Maasai traditional fermented milk products in Kenya. Curr Microbiol 2008;56: 315–321. 18175177 10.1007/s00284-007-9084-6

[2] Noori A Keshavarzian F Mahmoudi S Yousefi M Nateghi L Comparison of traditional Doogh (yogurt drinking) and Kashk characteristics (Two traditional Iranian dairy products). Eur J Exp Biol 2013;3: 252–255.

[3] Haghshenas B Haghshenas M Abdullah N Rosli R Radiah D Bioactivity characterization of Lactobacillus strains isolated from dairy products. Microbiologyopen 2015;4: 803–813. 26219634 10.1002/mbo3.280 PMC4618612

[4] Gueimonde M Delgado S Mayo B Ruas-Madiedo P Margolles A de los Reyes-Gavilán CG Viability and diversity of probiotic *Lactobacillus* and *Bifidobacterium* populations included in commercial fermented milks. Food Res Int 2004;37: 839–850.

[5] Singh S Goswami P Singh R Heller KJ Application of molecular identification tools for *Lactobacillus*, with a focus on discrimination between closely related species: A review. LWT - Food Sci Technol 2009;42: 448–457.

[6] Mirzaei H Barzgari A Isolation and molecular study of potentially probiotic lactobacilli in traditional white cheese of Tabriz in Iran. Ann Biological Res 2012;3: 2213–2216.

[7] Haghshenas B Nami Y Abdullah N Radiah D Rosli R Khosroushahi AY Anti-proliferative effects of *Enterococcus* strains isolated from fermented dairy products on different cancer cell lines. J Func Foods 2014;11: 363–374.

[8] Haghshenas B Nami Y Abdullah N Radiah D Rosli R Barzegari A Yari Khosroushahi A Potentially probiotic acetic acid bacteria isolation and identification from traditional dairies microbiota. Int J Food Sci Technol 2015;50: 1056–1064.

[9] Nami Y Abdullah N Haghshenas B Radiah D Rosli R Khosroushahi AY Probiotic assessment of *Enterococcus durans* 6HL and *Lactococcus lactis* 2HL isolated from vaginal microflora. J Med Microbiol 2014;63: 1044–1051. 24913559 10.1099/jmm.0.074161-0

[10] Haghshenas B Haghshenas M Nami Y Khosroushahi AY Abdullah N Barzegari A Probiotic assessment of *Lactobacillus plantarum* 15HN and *Enterococcus mundtii* 50H isolated from traditional dairies microbiota. Adv Pharm Bull 2016; 6: 37–47. 27123416 10.15171/apb.2016.07 PMC4845554

[11] Lim YS 2010 Probiotic Characteristics of bacteriocinogenic *Lactobacillus plantarum* strains isolated from malaysian food, biotechnology and biomolecular science. Phd thesis, Universiti Putra Malaysia (UPM), Kuala Lumpur.

[12] Deng W Xi D Mao H Wanapat M The use of molecular techniques based on ribosomal RNA and DNA for rumen microbial ecosystem studies: A review. Mol Biol Rep 2008; 35:265–274. 17484038 10.1007/s11033-007-9079-1

[13] Ben Amor K Vaughan EE de Vos WM Advanced molecular tools for the identification of lactic acid bacteria. J Nutr 2007;137: 741S–747S. 17311970 10.1093/jn/137.3.741S

[14] Pogačić T Mancini A Santarelli M Bottari B Lazzi C Neviani E Gatti M Diversity and dynamic of lactic acid bacteria strains during aging of a long ripened hard cheese produced from raw milk and undefined natural starter. Food Microbiol 2013;36: 207–215. 24010599 10.1016/j.fm.2013.05.009

[15] Liu CJ Gong FM Li XR Li HY Zhang ZH Feng Y Nagano H Natural populations of lactic acid bacteria in douchi from Yunnan Province, China. J Zhejiang Univ Sci B 2012;13: 298–306. 22467371 10.1631/jzus.B1100221 PMC3323945

[16] Doulgeraki AI Pramateftaki P Argyri AA Nychas G-JE Tassou CC Panagou EZ Molecular characterization of lactic acid bacteria isolated from industrially fermented Greek table olives. LWT - Food Sci Technol 2013;50: 353–356.

[17] Albufera U Bhugaloo-Vial P Issack MI Jaufeerally-Fakim Y Molecular characterization of *Salmonella* isolates by REP-PCR and RAPD analysis. Infect Genet Evol 2009;9: 322–327. 18243815 10.1016/j.meegid.2007.12.003

[18] De Vuyst L Camu N De Winter T Vandemeulebroecke K Van de Perre V Vancanneyt M Validation of the (GTG)_5_-rep-PCR fingerprinting technique for rapid classification and identification of acetic acid bacteria, with a focus on isolates from Ghanaian fermented cocoa beans. Int J Food Microbiol 2008;125: 79–90. 17920717 10.1016/j.ijfoodmicro.2007.02.030

[19] Ben Salah R Trabelsi I Ben Mansour R Lassoued S Chouayekh H Bejar S A new *Lactobacillus plantarum* strain, TN8, from the gastro intestinal tract of poultry induces high cytokine production. Anaerobe 2012;18: 436–444. 22634330 10.1016/j.anaerobe.2012.05.001

[20] Sahadeva RPK Leong SF Chua KH Tan CH Chan HY Tong EV Survival of commercial probiotic strains to pH and bile. Int Food Res J 2011;18: 1515–1522.

[21] Bhardwaj A Gupta H Kapila S Kaur G Vij S Malik RK Safety assessment and evaluation of probiotic potential of bacteriocinogenic *Enterococcus faecium* KH 24 strain under *in vitro* and *in vivo* conditions. Int J Food Microbiol 2010;141: 156–164. 20570005 10.1016/j.ijfoodmicro.2010.05.001

[22] Ahmadova A Todorov SD Hadji-Sfaxi I Choiset Y Rabesona H Messaoudi S Antimicrobial and antifungal activities of *Lactobacillus curvatus* strain isolated from homemade Azerbaijani cheese. Anaerobe 2013;20:42–49. 23357316 10.1016/j.anaerobe.2013.01.003

[23] Temmerman R Pot B Huys G Swings J Identification and antibiotic susceptibility of bacterial isolates from probiotic products. Int J Food Microbiol 2003;81: 1–10. 12423913 10.1016/s0168-1605(02)00162-9

[24] Munoz C Hidalgo C Zapata M Jeison D Riquelme C Rivas M Use of cellulolytic marine bacteria for enzymatic pretreatment in microalgal biogas production. Appl Environ Microbiol 2014;80: 4199–4206. 24795376 10.1128/AEM.00827-14 PMC4068657

[25] Dubey JP Choudhary S Kwok OC Ferreira LR Oliveira S Verma SK Isolation and genetic characterization of *Toxoplasma gondii* from mute swan (Cygnus olor) from the USA. Vet Parasitol 2013;195: 42–46. 23394800 10.1016/j.vetpar.2012.12.051

